# Genetic and Morphological Variation in Hypodontia of Maxillary Lateral Incisors

**DOI:** 10.3390/genes14010231

**Published:** 2023-01-16

**Authors:** Bernadette Kerekes-Máthé, Krisztina Mártha, Claudia Bănescu, Matthew Brook O’Donnell, Alan H. Brook

**Affiliations:** 1 Department of Morphology of Teeth and Dental Arches, Faculty of Dentistry, George Emil Palade University of Medicine, Pharmacy, Science and Technology of Targu-Mures, 540142 Targu-Mures, Romania; 2 Department of Orthodontics, Faculty of Dentistry, George Emil Palade University of Medicine, Pharmacy, Science and Technology of Targu-Mures, 540142 Targu-Mures, Romania; 3 Genetics Laboratory, Center for Advanced Medical and Pharmaceutical Research, George Emil Palade University of Medicine, Pharmacy, Science and Technology of Targu-Mures, 540142 Targu-Mures, Romania; 4 Annenberg School of Communication, University of Pennsylvania, Philadelphia, PA 19104, USA; 5 School of Dentistry, University of Adelaide, Adelaide, SA 5005, Australia; 6 Dental Institute, Barts and the London Medical Faculty, Queen Mary University of London, London E1 4NS, UK

**Keywords:** maxillary lateral incisor, hypodontia, *MSX1* gene, morphometrics

## Abstract

(1) Background: Hypodontia has a multifactorial aetiology, in which genetic factors are a major component. Associated with this congenital absence, the formed teeth may show differences in size and shape, which may vary with the specific genetic variants and with the location of the missing teeth. The aims of the present study were to investigate a specific variant of *MSX1*, derive morphometric tooth measurements in a sample of patients with isolated maxillary lateral incisor agenesis and matched controls, and model the findings. (2) Methods: Genotyping of the *MSX1* rs8670 genetic variant and morphometric measurements with a 2D image analysis method were performed for 26 hypodontia patients and 26 matched controls. (3) Results: The risk of upper lateral incisor agenesis was 6.9 times higher when the T allele was present. The morphometric parameters showed significant differences between hypodontia patients and controls and between the unilateral and bilateral agenesis cases. The most affected crown dimension in the hypodontia patients was the bucco-lingual dimension. In crown shape there was significant variation the Carabelli trait in upper first molars. (4) Conclusions: The *MSX1* rs8670 variant was associated with variations in morphological outcomes. The new findings for compensatory interactions between the maxillary incisors indicate that epigenetic and environmental factors interact with this genetic variant. A single-level directional complex interactive network model incorporates the variations seen in this study.

## 1. Introduction

Hypodontia, tooth agenesis, is a frequent variation of dental development. It may occur as part of a syndrome or in nonsyndromic form [1,2]. Third molars, upper lateral incisors and lower and upper second premolars are reported to be the teeth most frequently missing [3]. The prevalence of hypodontia is population-dependent and may be increasing [4]. Approximately 25% of individuals have congenitally missing third molars and 3.5% to 7% of the population have hypodontia of other permanent teeth, with females more frequently affected than males [5,6,7,8,9,10].

In maxillary lateral incisor agenesis, Bassiouny et al. [11] reported that 4.9% of an orthodontic group were affected. Other studies performed on different population samples found lower prevalence values of between 1.15% and 2.4% [12,13,14], while a study of twins reported a prevalence of 2.5% [15]. Bilateral agenesis of upper lateral incisors appears to be more common in some populations than unilateral agenesis [3], while other populations show a higher prevalence of unilateral cases [12,13]. Hypodontia of maxillary lateral incisors can be present alone, as a mild form of hypodontia (one or two missing teeth), or associated with missing premolars, in moderate forms of hypodontia (three to five missing teeth) or as part of many missing teeth, including molars, in severe forms of hypodontia (six or more missing teeth) [16,17]. Upper lateral incisor agenesis has a marked impact on the individual’s aesthetics, function, and psychosocial well-being [18], and is the focus of the present study. Pinho et al. suggest that upper lateral incisor agenesis may be a distinct kind of hypodontia [19].

Several studies have provided evidence that hypodontia has a multifactorial aetiology involving interactions between genetic, epigenetic, and environmental factors [18,20,21,22,23,24]. Using advances in Complexity and Network Science the interacting factors within network structures can be examined to increase understanding of complex biological systems [25]. Networks consist of nodes, which are connected by links, to form a web of interactions [26]. They follow organisational principles that make them functional. The network structure of a complex system contains information about its function and biological interactions [25], so that Network Science goes beyond structure and aims to identify the effects of networks on biological processes [27]. These networks can be single layer or multilayer. Within single layer networks the interactions can be undirected, or directed when the interactions lead to progressive development [25]. In weighted networks, different interactions have different intensities, while bipartite networks are formed by two distinct types of networks [27]. 

The influence of genetic factors in hypodontia has been demonstrated by many studies. The isolated agenesis of maxillary lateral incisors may sometimes be transmitted as an autosomal dominant characteristic, with variable expression and incomplete penetrance [9,28]. Frequently studied genes in relation to maxillary lateral incisor agenesis are *MSX1* (muscle segment homeobox 1), *PAX9* (paired box gene 9), *AXIN2* (axis inhibition protein 2), and *EDA* (ectodysplasin A) [24,29]. *MSX1* is expressed in regions of condensing ectomesenchyme in the tooth germ, and *MSX1* gene polymorphisms have been associated with various forms of hypodontia in humans. The homeodomain of *MSX1* stimulates apoptosis of cancer cells [19], which might offer a role for the *MSX1*, as an indicator of tumor susceptibility [30]. The *MSX1* rs8670 genetic variant, known as transition *6C > T polymorphism (see dbSNP database: http://www.ncbi.nlm.nih.gov/snp, accessed on 10 February 2022), may contribute to the aetiology of hypodontia of maxillary lateral incisors [31] and is investigated in the present study. 

Hypodontia has been associated with several dental and skeletal features, including variations in tooth size and shape, dental arch and craniofacial morphology [9,11,32,33,34,35]. The phenotype of isolated hypodontia of the maxillary lateral incisors includes changes in size and shape of the other teeth, including the antimere in patients with unilateral hypodontia of upper lateral incisors. Within an individual with hypodontia, the variations in the dimensions have given rise to the concept of localised ‘compensatory interactions’ [9,15]. Sofaer et al. proposed that the growth potential of a developing tooth is increased if there is more space available [36]. Investigating this theory, subsequent studies have explored compensatory interactions with different methodologies and results. Kondo and Hanamura stated that in multiple congenitally absent teeth, the size of the formed teeth is reduced, but in mild hypodontia the formed teeth are larger than in controls [37]. A twin study investigating possible compensatory interactions between developing maxillary anterior teeth confirmed that developmental variations of lateral incisors are associated with morphogenetic variations of the adjacent teeth, and that environmental factors influence the Complex Adaptive System of dental development during the later stages of morphogenesis [15].

The aims of the present study were to investigate specific candidate genes and morphometric measurements in a sample of patients with isolated maxillary lateral incisor agenesis and matched controls to (1) gain further knowledge of the effects of the interacting factors in the aetiology of this dental variation, (2) explore further the interactions during development of these teeth, and (3) consider how these findings can be modelled.

## 2. Materials and Methods

This study was approved by the Ethics Committee of Scientific Research of the George Emil Palade University of Medicine, Pharmacy, Science and Technology of Targu Mures (Approval no. 60/07.03.2018).

### 2.1. Sampling

Twenty-six patients with upper lateral incisor agenesis, 13 females and 13 males, aged between 12 and 24 years were included. Prior to examination, informed consent was obtained from each subject. The criteria for inclusion were the unilateral or bilateral congenital absence of the permanent upper lateral incisor, and that the formed permanent teeth were fully erupted. Diagnosis was based on dental history, clinical examination, and orthopantomographic radiographs. Exclusion criteria were the presence of any other congenitally absent teeth or any other congenital conditions, syndromes, or a history of orthodontic treatment or tooth extraction. The same number of controls with complete permanent dentitions, matched for sex, age, and ethnicity (ethnic Romanian) were also included from the same dental clinic from individuals who presented for dental screening and did not present tooth agenesis, syndromes, orthodontic treatment or tooth extraction. 

### 2.2. Genotyping

Epithelial cells from the oral mucosa were obtained using ethylene oxide treated buccal swabs (Isohelix DNA Buccal Swabs, Isohelix Ltd., Kent, UK). Tubes were coded and transported for DNA isolation and genotyping to the Center for Advanced Medical and Pharmaceutical Research of The University of Medicine, Pharmacy Science and Technology from Targu Mures. DNA obtained from the specimens was quantified using the Eppendorf BioSpectrometer basic system and stored at −20 °C until they were genotyped. For the present study one single nucleotide polymorphism of the *MSX1* gene was selected for genotyping. Genotyping of the *MSX1* rs8670 genetic variant was performed using the corresponding pre-design TaqMan SNP Genotyping Assay on a 7500 Fast Dx Real-Time PCR Instrument.

### 2.3. Imaging and Morphometrics

Alginate impressions (Ypeen Premium, SpofaDental, Jičín, Czech Republic) were taken from each individual and study models were poured in dental stone (FujiRock, GC). Images of the study models were captured by a digital DSLR camera (Nikon D3100, Nikon Corporation, Tokyo, Japan) with a macro lens (Tamron SP AF-S 90 mm f/2.8). The camera was fixed above the dental cast, on an adjustable stand (Kaiser 5360, Kaiser Fototechnik, Buchen, Germany), with two LED bulbs providing standard lighting conditions. Images were then processed by Image Pro Insight 9.3 software (Media Cybernetics, Rockville, MD, USA), using a validated 2D image analysis method [38]. Each image taken included a ten-millimeter scale for calibration and the measurements were made directly on the images. The measured parameters are given in Table 1. Intraoperator reproducibility was determined using the upper and lower models of eight individuals. 

**Table 1:** Description of the morphometric measurements.

Type of Measurement	Description
Mesio-distal dimension	The greatest distance between the mesial and distal contact points (Figure 1).
Bucco-lingual dimension	The greatest distance of the tooth between the buccal and the lingual surface (Figure 1).
Occlusal area	Area of the occlusal surface defined by the mesial, buccal, distal, and lingual margins (Figure 1).
Intercuspal distance on upper first and second premolars	Distance between the tips of the buccal and lingual cusps.
Intercuspal distance on lower first premolars	Distance between the tips of the buccal and lingual cusps (Figure 2).
Intercuspal distance on lower second premolars	Distance between the tips of the two lingual cusps measured only in the case of the three-cusp version of the tooth (Figure 2).
Intercuspal distances on upper first molars	Distance between the tips of the cusps: mesiobuccal to distobuccal (MB-DB); distobuccal to mesiolingual (DB-ML); mesiolingual to mesiobuccal (ML-MB); and mesiobuccal to distolingual (MB-DL) (Figure 3).
Size of the cusp Carabelli on the upper first permanent molars	Diagnosed using the Arizona State University Dental Anthropology System [39]

### 2.4. Statistical Analysis

Data were entered on Microsoft Excel sheets and statistical analysis was performed using MedCalc (MedCalc Software Ltd., Ostend, Belgium). The chi-square test with Yates correction was used for comparison of the studied genotype between the two groups, and odds ratio was calculated. The confidence level was established at 95%. Regarding the morphometric parameters, outliers were excluded, and normal distribution of the data was confirmed (Shapiro-Wilk test of normality). Inferential statistics were performed using Intraclass Correlation Coefficients (ICC), Fisher’s exact test, one-way ANOVA and unpaired t-tests. The significance level was set to 0.05.

## 3. Results

In 58% of the cases, unilateral agenesis of the lateral incisor was found, while in 42% of the cases the agenesis was bilateral. Eight females and seven males presented unilateral agenesis, while five females and six males had bilateral agenesis (*p* = 0.69). The difference between the side of unilateral agenesis was not significant statistically (*p* = 0.54).

Significant differences in the distribution of *MSX1* rs8670 genetic variant alleles were found. The association between the variant type genotype (C/T + T/T) and the risk of developing upper lateral incisor agenesis was significant. The frequency of the variant T alleles was significantly higher in the hypodontia group. The risk of upper lateral incisor agenesis was 6.9 times higher when the T allele was present (Table 2). In bilateral agenesis the presence of the T allele was more frequent, although this difference was not significant statistically (*p* = 0.07). The distribution of *MSX1* rs8670 genotypes was in accordance with Hardy-Weinberg equilibrium for upper lateral incisor agenesis (*p* = 0.195) and also for controls (*p* = 0.670).

The intra-operator reliability of the morphometric measurements was excellent, with all ICC values being higher than 0.8.

Mesiodistal (MD) and buccolingual (BL) diameters from the occlusal view, as well as occlusal surface area (OA), were measured for all teeth. No significant differences between antimere teeth were found; therefore, results were averaged for tooth pairs. 

Lower central and lateral incisors, upper first premolars and lower second premolars showed significantly smaller MD diameters in hypodontia patients than in the control group (Table 3). Buccolingual diameters showed significant differences in the case of the upper and lower first incisors, upper premolars, lower canines, lower second premolars, and lower molars, which were smaller in the hypodontia group than in controls. Upper first premolars, lower central incisors, and lower second premolars showed significant differences in all three general measurement types (Table 3). In the upper first incisors, almost all values were significantly smaller in the hypodontia group than in controls. 

To investigate compensatory interactions between maxillary central and lateral incisors, data for the unilateral and bilateral hypodontia patients are included in a separate table (Table 4). This shows that in bilateral agenesis of the upper lateral incisor, mesio-distal and bucco-lingual diameters of the central incisors, measured from the occlusal view, were significantly smaller than in controls. In unilateral agenesis where the other lateral incisor was present with average dimensions, the mesio-distal dimension of the central incisors was slightly larger than in controls (Table 4).

Intercuspal distances in all premolars showed significantly smaller dimensions in the maxillary lateral incisor agenesis group than in controls (Table 5). The greatest differences were present in lower second premolars: premolars with three cuspshad significantly smaller intercuspal distances between the lingual cusps in the hypodontia group than in controls (*p* < 0.00001). Regarding the intercuspal distances in the upper first molars, no statistically significant differences were detected between the hypodontia group and the controls (Table 5). No differences between sexes regarding intercuspal distances were detected.

Cusp numbers on premolar and first molar teeth showed moderate differences between the groups. Slightly more lower second premolars with three cusps in controls were detected, but this difference was not significant (*p* > 0.07). In lower first premolars a few with three cusps in the control group were detected, but the difference was not significant (*p =* 0.19). Lower first molars also showed a slight variation in cusp number, some having only four cusps in each group, without significant differences (*p =* 0.76). Upper first and second premolars, as well as upper first molars, showed no variation in cusp number, either in the hypodontia group or the control group. In upper first molars smaller (size 0–4), Carabelli cusps were present in the case of the upper lateral incisor agenesis group than in controls (*p =* 0.003; OR = 12.89), and it was more likely no Carabelli trait was present than in controls (*p =* 0.0005; OR = 6.74).

When comparing results between patients with unilateral and bilateral maxillary incisor agenesis, significant differences were detected only in the mesio-distal dimension of upper central incisors, upper first premolars, lower lateral incisors, lower second premolars, and lower first molars. The mesio-distal dimension of all these teeth was smaller in the bilateral agenesis group than in the unilateral agenesis group. Significant results are shown in Table 6.

Multivariate analysis of variance was performed to determine the effect of the *MSX1* rs8670 alleles and the effect of the hypodontia on different parameters. Upper central incisor parameters as the mesial adjacent teeth, and the intercuspal distance of lower second premolars, as the most affected intercuspal distance, were explored. The effect of the gene factor was strong in case of the parameters of the upper central incisors, while the effect of the presence of unilateral or bilateral hypodontia or the absence of hypodontia was strong in case of the intercuspal distance of the lower second premolar (Table 7).

## 4. Discussion

Different polymorphisms of the *MSX1* gene may result in different phenotypic outcomes from dental development. In this investigation the *MSX1* rs8670 variant was studied. According to Ensembl the frequency of the variant allele rs 8670 in the *MSX1* gene is 19% in all individuals, with differences between populations as follows: 23% in Europeans, 16% in Americans, 26% in Africans, 6% in East Asians, 21% in South Asians (https://www.ensembl.org, accessed on 28 December 2022).

A recent study from Poland investigated the association between *MSX1* gene polymorphisms and impacted teeth. A large control group was used, and the frequency of the *MSX1* rs 8670 polymorphisms was similar to the frequency found in the control group of the present study. The authors performed a haplotype analysis of *MSX1* rs8670 and rs12532 polymorphisms, which showed no differences between controls and cases with impacted teeth. They found a 1.6 times lower frequency of the *MSX1* (C, G) haplotype in the subgroups of cases with a total number of impacted teeth below and above the median value [40].

Liang J. et al. [41] found that, depending on their location in the gene, variants of *MSX1* gene cause different phenotypes. Variants affecting the homeodomain are associated with tooth agenesis. They investigated 31 *MSX1* variants causing tooth agenesis with or without other phenotypes, as well as different syndromes [41]. *MSX1* rs8670 is a 3′ UTR variant. The 3′ UTR region has an important role in the control of gene expression [42] and 3′ UTR polymorphisms may affect the 3′ UTR regulation by influencing the post-transcriptional regulation and the stability of the mRNA [31].

In the evolving landscape of genetic factors in hypodontia some 20 different genes have been shown to be associated with non-syndromic tooth agenesis [43,44]. Within this landscape, Martha K. et al. [45] showed that the variant genotype of the *MSX1* rs8670 was more frequent in patients with agenesis of anterior teeth and increased the risk of hypodontia in the studied population (OR = 6.6). They also found that this genotype was less frequent in other types of hypodontia. The present study investigated a similar population and, therefore, *MSX1* was examined in this study. Boeira et al. found that the allele frequency of the T allele of *MSX1* was significantly higher in a group of individuals with common hypodontia phenotypes compared to a control group without hypodontia, demonstrating the relationship between *MSX1* rs8670 and hypodontia in their sample [31]. In the present study, the frequency of the T allele was significantly higher in the hypodontia group than in controls, confirming the role of this polymorphism in a number of patients with isolated maxillary lateral incisor agenesis.

This study has added new knowledge to the debate concerning whether compensatory interactions occur during tooth development in patients with maxillary incisor hypodontia. The original theory of Sofaer et al. [36] states that in the agenesis of maxillary lateral incisors, the central incisor adjacent to the missing tooth will be slightly larger. When the agenesis is unilateral, the adjacent central incisor will be larger than its antimere, and when the agenesis is bilateral, both central incisors will be larger than average [36]. This concept was incorporated by Brook as a component of his unifying aetiological model of dental variations having a multifactorial background [5]. Different studies on this topic, using different methodologies, have shown varying results. While Kieser et al. [46] concluded that there is no evidence of these compensatory interactions in the dentition [46], Kondo and Hanamura [37] found evidence that the formed adjacent teeth can be larger than in controls, and Tadros et al. [15] found evidence of compensatory interactions in twins [15]. The present study provides new evidence, finding different results in patients with unilateral and bilateral agenesis of maxillary lateral incisors. In patients with unilateral agenesis, where the other lateral incisor was present with average dimensions, the mesio-distal dimensions of the central incisors were slightly larger than the controls. When a microdont lateral incisor was present and the other was missing, the mesio-distal dimension of the central incisor was slightly smaller than in controls, with the bucco-lingual dimension of central incisors significantly smaller than in controls. In bilateral agenesis of the upper lateral incisor, mesio-distal and bucco-lingual diameters of the central incisors, measured from the occlusal view, were significantly smaller than in controls. These findings of a range of phenotypic outcomes refine the concept of compensatory interactions, reflecting the multiple factors and variable interactions involved during development. These factors can be modelled as agents within a complex interactive network of genetic, epigenetic and environmental factors. Each factor will have a different weight in the dental development of different individuals.

The next consideration is whether the factors causing isolated maxillary lateral incisor agenesis have an effect on the development of teeth other than maxillary incisors. Wright et al. [33] found that in hypodontia of the maxillary lateral incisors the other teeth in the dentition were smaller than those of controls. There was a similar result in the present study, but with the additional finding that there was variation in the degree to which each tooth type was affected in size and shape, including cusp numbers and intercuspal distances. The findings are predominantly in accordance with developmental timing [47] and the Morphological Field Model [48,49] with the key, early forming teeth of each tooth type being least affected. Thus, the upper first molars, as key teeth in their class, showed no variation in diameters, area, intercuspal distances, or cusp numbers. The only significant variation in the upper first molars was the Carabelli trait.

The most affected crown dimension in the hypodontia patients was the bucco-lingual dimension, which was smaller in the hypodontia group than in controls, with most of the differences being significant. The occlusal area measurements were also smaller in the hypodontia patients but to a lesser degree than bucco-lingual. Mesio-distal measurements were smaller in hypodontia than in controls, but to a lesser extent than bucco-lingual or occlusal. The most affected teeth in the lower arch were the central incisors and the second premolars, while in the upper arch the first premolars were most affected. The least affected teeth in the lower arch were the first premolars, and in the upper arch were the canines and the first molars. Compatible findings are present in the literature [34,50]. These different effects of hypodontia on the detailed morphology of the dentition again reflect the complexity of dental development.

Sexual dimorphism may be an important factor. In an epidemiological population study, Brook [5] found that, overall, females more frequently had tooth agenesis than males. Some published studies suggest a possible sexual dimorphism of tooth agenesis patterns in specific locations within the dentition. A study conducted in Japan showed a significantly higher prevalence of lower central incisor agenesis in males than in females. This study was conducted on orthodontic patients with non-syndromic oligodontia and found sexual dimorphism of the location of the tooth agenesis. The authors do not exclude the possibility of biased results by the more frequent orthodontic treatment of females than males for tooth agenesis [51]. Another study, conducted on non-syndromic, white European individuals, investigated third molar agenesis patterns. They found no sexual dimorphism regarding the patterns of third molar agenesis. They also found that when agenesis of other teeth is present, third molar agenesis is more frequently present, both in females and males, without significant pattern differences [52]. Another study included all types of severity groups of tooth agenesis and explored the patterns by gender in a large Austrian population. The most common agenesis patterns were found in the mild hypodontia group. Individuals with milder forms of hypodontia showed a higher degree of similar agenesis patterns than individuals with more severe forms of agenesis, which means that the similarity of agenesis patterns decreased as the number of congenitally missing teeth increased [53]. The present study includes only individuals with mild hypodontia: congenitally missing upper lateral incisors. No sexual dimorphism was detected in the studied group regarding agenesis patterns of the upper lateral incisors.

While polymorphic variants contribute to the genetic component of the aetiology of hypodontia, their contribution to the complex aetiology is often modest. The investigation of the single variant *MSX1* rs 8670 limits the degree to which the findings of this study can be generalized to other variants and association studies. Although the size of the sample fulfilled the requirements of the statistical power calculation, the numbers in some components of the investigation were small for a genetic study. This is a limitation even though the association found confirms previous reports.

The final part of this discussion considers the third aim, how this existing and new knowledge can be modelled to enhance understanding of dental development. There is variation involving tooth number, size, and shape. Underlying factors include genetics, e.g., *MSX1* variants, and epigenetic/environmental effects as seen in compensatory interactions. These factors have complex effects, with different outcomes for the three spatial dimensions of the tooth crown and for cuspal patterns. This complexity is reflected in the different emphases of the models that have been proposed based on phenotype or patterning or molecular genetics. The findings of this study are compatible with the phenotypic quasi-continuous model proposed, and then further developed, by Brook [5,32]. The patterns seen also accord with the morphogenetic field model of Butler [54] and Dahlberg [48]. A co-operative genetic interaction model proposed by Mitsiadis and Smith [55], which was extended by Townsend et al. [56], incorporated the homeobox gene concept [57], the morphogenetic field model, and the clone theory of Osborne [58]. However, the co-operative genetic interaction model does not account for the epigenetic/environmental compensatory interactions seen in this study, nor does the inhibitory cascade model of Jernvall [59] and Salazar-Cuidad and Jernvall [60].

Therefore, a multiple model approach is suggested which combines the insights of each of the different models and approaches. In addition to the previous models, here it is proposed that a single level directional complex interactive network approach incorporates the genetic and environmental variations seen in this study.

## 5. Conclusions

In relation to the three aims of the study:

(1) The genetic factor *MSX1* rs8670 variant was associated with variable phenotypic outcomes in multiple morphological parameters of the formed teeth.
(2) The new findings concerning compensatory interactions in the maxillary incisor region indicate interactions of epigenetic and environmental factors with the genetic variant.
(3) A multiple model approach increases understanding of the aetiology of tooth agenesis. The addition of a single level, directional, complex interactive approach incorporates the findings of this study.

## Figures and Tables

**Figure 1:**
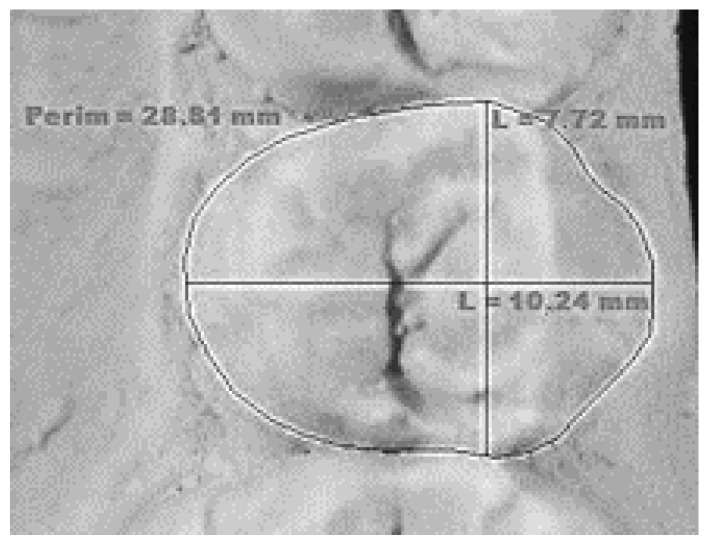
Mesio-distal and bucco-lingual diameter and area measurement presented on an upper premolar.

**Figure 2:**
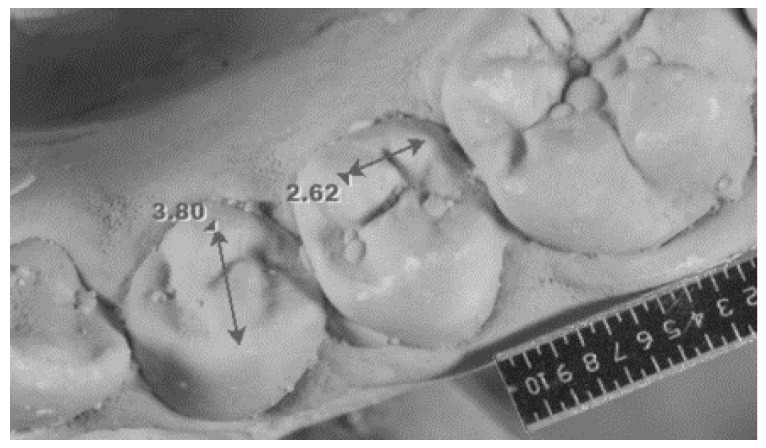
Intercuspal distance measurement on lower premolars.

**Figure 3:**
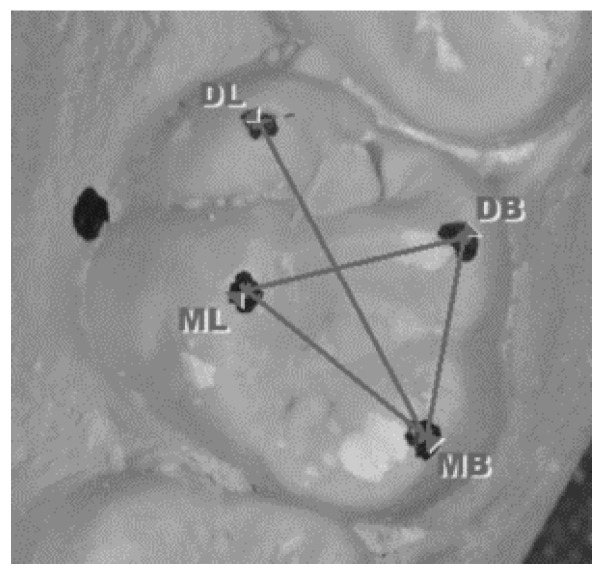
Intercuspal distances measured on an upper first molar.

**Table 2:** Genotype and allele frequencies of *MSX1* rs8670 genetic variant in upper lateral incisor agenesis.

*MSX1* rs8670 C/T	Upper Lateral Incisor Agenesis (N = 26) n	Matched Controls (N = 26) n	Statistical Analysis for Genotypes	Statistical Analysis for Alleles
C/C	12	22	C/T, T/T vs. C/C ***p = 0.007*** OR = 6.41	T vs. C ***p = 0.0003*** OR = 6.90
C/T	9	4		
T/T	5	0		
C	33	48		
T	19	4		

OR = odds ratio; C = wild type allele; T = variant (minor) allele. Bold values denote statistical significance at the *p* < 0.05 level.

**Table 3:** Mean values and *p* values for statistical significance for the measured tooth dimensions.

Teeth	MD			BL			OA		
	Hypodontia	Control	*p*	Hypodontia	Control	*p*	Hypodontia	Control	*p*
1.1; 2.1	8.63 ± 0.53	8.87 ± 0.6	*0.06*	6.65 ± 0.74	7.45 ± 0.62	***0.01***	44.63 ± 6.37	51.51 ± 8.37	***0.03***
1.3; 2.3	7.78 ± 0.59	7.82 ± 0.47	*0.81*	7.97 ± 0.63	8.13 ± 0.26	*0.43*	44.76 ± 5.35	46.99 ± 4.12	*0.33*
1.4; 2.4	6.99 ± 0.54	7.27 ± 0.48	***0.009***	9.07 ± 0.39	9.61 ± 0.68	***0.01***	50.56 ± 3.98	55.88 ± 7.83	***0.03***
1.5; 2.5	6.70 ± 0.37	6.82 ± 0.48	*0.42*	9.04 ± 0.62	9.74 ± 0.60	***0.01***	47.53 ± 5.89	53.17 ± 8.18	*0.10*
1.6; 2.6	10.36 ± 0.56	10.48 ± 0.54	*0.42*	11.00 ± 0.27	11.97 ± 0.61	*0.81*	99.49 ± 10.26	106.20 ± 11.86	*0.15*
3.1; 4.1	5.40 ± 0.4	5.67 ± 0.44	***0.03***	5.77 ± 0.40	6.27 ± 0.41	***0.01***	22.01 ± 1.49	26.00 ± 3.80	***0.004***
3.2; 4.2	5.83 ± 0.45	6.12 ± 0.39	***0.02***	6.10 ± 0.57	6.52 ± 0.43	*0.08*	24.94 ± 2.13	28.90 ± 3.62	***0.005***
3.3; 4.3	6.77 ± 0.48	6.88 ± 0.46	*0.43*	6.89 ± 0.90	7.62 ± 0.36	***0.03***	33.99 ± 4.67	38.10 ± 3.68	***0.03***
3.4; 4.4	7.17 ± 0.34	7.10 ± 0.47	*0.78*	8.02 ± 0.57	8.05 ± 0.53	*0.86*	42.57 ± 3.31	45.37 ± 5.68	*0.11*
3.5; 4.5	7.03 ± 0.45	7.31 ± 0.45	***0.04***	8.36 ± 0.54	8.75 ± 0.57	***0.04***	45.55 ± 5.13	50.46 ± 5.87	***0.03***
3.6; 4.6	11.05 ± 0.75	11.20 ± 0.67	*0.45*	10.50 ± 0.43	11.13 ± 0.65	***0.01***	96.72 ± 8.42	105.05 ± 10.60	*0.06*

MD = mesio-distal; BL = bucco-lingual; OA = occlusal area. Bold values denote statistical significance at the *p* < 0.05 level.

**Table 4:** Mean values of upper central incisors and upper lateral incisors (where they were present).

**MD**						
**Teeth**	1.1; 2.1			1.2 or 2.2		
	Hypodontia	Control	*p*	Hypodontia	Control	*p*
**A**	8.93 ± 0.65	8.86 ± 0.57	*0.69*	6.27 ± 0.69	6.96 ± 0.58	***0.02***
**B**	8.79 ± 0.45		*0.68*	4.82 ± 0.54		***<0.0001***
**C**	8.86 ± 0.53		*0.98*	5.59 ± 0.96		***0.0001***
**D**	8.35 ± 0.31		***0.01***	NA		NA
**BL**						
**Teeth**	1.1; 2.1			1.2 or 2.2		
	Hypodontia	Control	*p*	Hypodontia	Control	*p*
**A**	6.84 ± 0.53	7.45 ± 0.62	*0.11*	6.37 ± 0.87	6.47 ± 0.68	*0.82*
**B**	6.50 ± 0.87		***0.04***	4.72 ± 0.49		***<0.001***
**C**	6.69 ± 0.67		***0.02***	5.60 ± 1.10		*0.08*
**D**	6.58 ± 0.94		***0.03***	NA		NA
**OA**						
**Teeth**	1.1; 2.1			1.2 or 2.2		
	Hypodontia	Control	*p*	Hypodontia	Control	*p*
**A**	46.98 ± 7.67	51.51 ± 8.37	*0.42*	32.02 ± 7.58	36.02 ± 2.62	*0.41*
**B**	43.48 ± 5.51		*0.09*	18.17 ± 2.82		***0.001***
**C**	45.42 ± 6.63		*0.12*	25.56 ± 9.13		***0.01***
**D**	43.20 ± 6.33		*0.12*	NA		NA

A = Unilateral hypodontia + average antimere lateral incisor (9 cases); B = Unilateral hypodontia + microdont antimere lateral incisor (6 cases); C = Unilateral hypodontia of upper lateral incisors regardless of the size of the antimere lateral incisor; D = Bilateral hypodontia of maxillary lateral incisors (11 cases). Bold values denote statistical significance at the *p* < 0.05 level.

**Table 5:** Mean intercuspal distances in premolars and upper first molar.

Intercuspal Distance	Upper Lateral Incisor Agenesis Group	Matched Controls	*p*
Upper first premolars	5.39 ± 0.59	5.88 ± 0.46	***0.03***
Upper second premolars	5.60 ± 0.39	6.04 ± 0.41	***0.0001***
Lower first premolars	3.63 ± 0.61	4.02 ± 0.53	***0.01***
Lower second premolars (distance between lingual cusps)	2.78 ± 0.39	3.54 ± 0.31	***<0.00001***
Upper first molars			
MB-DB	5.08 ± 0.49	5.04 ± 0.44	*0.38*
DB-ML	6.92 ± 0.77	6.73 ± 0.42	*0.14*
ML-MB	6.49 ± 0.53	6.57 ± 0.56	*0.31*
MB-DL	8.96 ± 0.47	9.24 ± 0.74	*0.07*

MB = mesio-buccal; DB = disto-buccal; ML = mesio-lingual; DL = disto-lingual. Bold values denote statistical significance at the *p* < 0.05 level.

**Table 6:** Mean values of mesio-distal dimension in case of teeth with significant differences.

Teeth	1.2; 2.1	1.4; 2.4	3.2; 4.2	3.5; 4.5	3.6; 4.6
Unilateral agenesis	8.86 ± 0.53	7.20 ± 0.58	6.09 ± 0.39	7.21 ± 0.51	11.36 ± 0.78
Bilateral agenesis	8.35 ± 0.31	6.74 ± 0.32	5.51 ± 0.25	6.83 ± 0.27	10.68 ± 0.48
*p* value	***0.01***	***0.01***	***0.0003***	***0.03***	***0.01***

Bold values denote statistical significance at the *p* < 0.05 level.

**Table 7:** Results of the multivariate analysis of variance regarding the effect of the gene factor and the hypodontia factor on relevant parameters.

Outcome Variables	Effect of the Gene Factor (T Allele of the rs8670 in the *MSX1* Gene) (N = 14)		Effect of the Hypodontia Factor (Unilateral/Bilateral Hypodontia/Control)	
	***p* value**	**F ratio**	***p* value**	**F ratio**
Mesio-distal diameter of upper central incisors	***0.04***	7.39	*0.29*	1.57
Bucco-lingual diameter of upper central incisors	***0.05***	6.61	*0.34*	1.31
Occlusal area of upper central incisors	***0.01***	13.03	*0.93*	0.03
Intercuspal distance of lower second premolars	*0.67*	0.20	***0.01***	11.49

Bold values denote statistical significance at the *p* < 0.05 level.

## Data Availability

Data available on request due to privacy restrictions.
